# Integrating Culture and History to Promote Health and Help Prevent Type 2 Diabetes in American Indian/Alaska Native Communities: Traditional Foods Have Become a Way to Talk About Health

**DOI:** 10.5888/pcd17.190213

**Published:** 2020-02-06

**Authors:** Lemyra DeBruyn, Lynne Fullerton, Dawn Satterfield, Melinda Frank

**Affiliations:** 1 Native Diabetes Wellness Program, Division of Diabetes Translation, Centers for Disease Control and Prevention, Albuquerque, New Mexico; 2 Department of Emergency Medicine, University of New Mexico Health Sciences Center, Albuquerque, New Mexico; 3 Native Diabetes Wellness Program, Division of Diabetes Translation, Centers for Disease Control and Prevention, Atlanta, Georgia

## Abstract

**Purpose and Objectives:**

The purpose of the Traditional Foods Project (TFP) was to implement and evaluate a community-defined set of strategies to address type 2 diabetes by focusing on traditional foods, physical activity, and social support. The TFP sought to answer 2 questions: first, how do we increase and sustain community access to traditional foods and related activities to promote health and help prevent type 2 diabetes? Second, how do we evaluate interventions across culturally and geographically diverse communities to demonstrate success?

**Intervention Approach:**

Public health interventions are most effective when communities integrate their own cultures and history into local programs. The food sovereignty movement among American Indians/Alaska Natives and indigenous populations globally offers ways to address public health issues such as chronic diseases like type 2 diabetes. Historical, economic, social, and environmental determinants of health are critical to understanding the disease.

**Evaluation Methods:**

During 2008–2014, seventeen tribal TFP partners implemented locally designed interventions and collected quantitative and qualitative data in 3 domains: traditional foods, physical activity, and social support. Partners entered data into a jointly developed evaluation tool and presented additional program data at TFP meetings. Partner observations about the effect of the TFP were gathered in planned discussions.

**Results:**

Quantitative results indicate collaborative community engagement and sustained interventions such as gardening, availability of healthy foods across venues, new health practices, health education, and storytelling. Qualitative results demonstrate the importance of tribally driven programs, underscoring the significance of traditional foods in relation to land, identity, food sovereignty, and food security.

**Implications for Public Health:**

Traditional foods and food sovereignty are important areas for American Indian/Alaska Native communities to address the public health issues of chronic disease, specifically type 2 diabetes, locally and nationwide.

**Summary**

**What is already known on this topic?**

Historical, economic, social, and environmental determinants of health are critical to understanding type 2 diabetes in American Indian and Alaska Native communities.

**What is added by this report?**

Integrating history and culture, 17 tribes and tribal organizations worked during 2008–2014 to increase and sustain community access to traditional foods to promote health and help prevent type 2 diabetes. In partnership with a federal program and each other, tribal partners evaluated community-based interventions locally and across their culturally and geographically diverse communities to demonstrate effectiveness.

**What are the implications for public health practice?**

Traditional healthy foods and food sovereignty are valuable areas for American Indian and Alaska Native communities to address chronic disease, specifically type 2 diabetes.

## Introduction

Diabetes is highly prevalent in the United States and is associated with increased risk of health problems such as vascular diseases (eg, heart disease, stroke), chronic kidney disease, and blindness (1). It is the seventh leading cause of death in the United States and affects more than 30 million Americans; an additional 84 million adults have prediabetes, and thus are at risk for diabetes (1). Although not all diabetes is preventable, type 2 diabetes, which accounts for 90% to 95% of all diabetes cases, can sometimes be prevented by maintaining a healthy diet, a healthy weight, and a healthy level of physical activity (2). The prevalence of diabetes among US adults has been stable for approximately 10 years, but this is not true of children, adolescents, and young adults (3). A study comparing the prevalence of diabetes among children, adolescents, and young adults (aged <20 y) from 2001 and 2009 found large increases in the prevalence of both type 1 (30.0%, from 1.48 to 1.93 per 1,000 persons) and type 2 (35.0%, from 0.34 to 0.46 per 1,000 persons) diabetes (3). Health complications in this age group are common. In a study of children and adolescents who had a diagnosis of diabetes for at least 5 years, data collected during 2011–2015 showed that 32% with type 1 and 72% with type 2 had at least 1 health complication related to the function of kidneys, eyes, the heart, and the nerve and circulatory systems (4).

Diabetes is not equally distributed among US racial and ethnic groups. The rate of diagnosed diabetes among American Indian/Alaska Native (AI/AN) adults in 2013–2015 (15.1%) was twice the rate among non-Hispanic white adults (7.4%) (5). Disparities among young people are greater. The incidence of type 2 diabetes among AI/AN children, adolescents, and young adults aged 10 to 19 years in 2011–2012 (46.5 per 100,000 population) was more than 10 times the incidence among their non-Hispanic white counterparts (3.9 per 100,000 population) (1). This disparity may be related in part to differences in obesity rates, a known diabetes risk factor. In 2015–2016, the prevalence of obesity among non-Hispanic white children, adolescents, and young adults aged 2 to 19 years in the United States was 14.1% (6). Among AI/ANs of the same age in 2015, the prevalence was 29.7% (7).

The history of type 2 diabetes in the United States illuminates complex issues. The transition from local, harvested foods to foods dense in calories and fat fueled rates of type 2 diabetes and related chronic conditions (8). After World War II, with the shift to a wage economy (9), Americans began to consume readily available processed foods high in sugar and fat and low in fiber and were typically less physically active than before. Rates of diabetes in the United States rose from less than 1% in 1958 (~500,000 people) to 9.4% in 2015 (30.3 million people) (1). Diabetes was also rare among AI/ANs before 1940. Among AI/ANs, as among other Americans, the dramatic changes in diet and declining physical activity preceded rising rates of the disease (8).

Focusing on biologic factors alone overlooks factors that propel development of chronic diseases (10–12). Recognizing historical, economic, and environmental contributions, or social determinants of health, is critical to understanding the trajectory of type 2 diabetes (13,14).

Current social determinants of health associated with development of type 2 diabetes include poverty (15), attaining less than a high school education (5,16), physiologic stress responses associated with historical trauma (17), and adverse childhood experiences (18–20). Food insecurity, defined as uncertain or limited access to enough food for a healthy life, is also correlated with increased risk of developing type 2 diabetes (21). Rates of food insecurity among AI/AN children are approximately 2 times national rates (22). In 2016 nearly 30% of AI/AN households were food insecure, compared with 16% of non-AI/AN households (23,24).

For tribal nations, gathering, planting, or hunting food was integral to physically active and spiritual lives (25). Decades of federal mandates affected the land and water resources of tribal nations, which in turn profoundly disrupted indigenous food systems and reduced access to traditional foods (9,13,25–32). Native peoples in the United States were forced to move and had to adjust to different lands, climates, and the foods they could raise and gather. These foods were often supplemented by government provisions to stave off starvation and malnutrition resulting from disrupted food systems (33,34).

Since the 1970s, federal food distribution programs have provided commodity foods to AI/AN communities (34). These processed foods, high in salt and fat, and demanding very little physical activity to access, often result in what Indian people call a “commod bod” (a “commodity body,” or a body type resulting from consuming commodity foods) (35). Furthermore, food assistance programs alone do not substantially improve food insecurity (24). Some traditional foods (bison, blue corn meal, wild rice) were added recently to food assistance programs, but these foods are not regularly available (34). Access to healthy food across Indian Country is further thwarted by distance (food deserts), limited transportation, inadequate supermarkets, environmental contamination, and little money to purchase healthy foods (31,33,34).

Tribally driven approaches to understanding these issues in Indian Country include indigenous science, sometimes called traditional ecological knowledge, a natural science grounded in lifetimes of observation, experimentation, and adaptation (36). A blueprint for a way of life that has survived (37), traditional ecological knowledge is inextricably linked to traditional foods and food sovereignty. It informs cultivating, harvesting, and sharing foods; storytelling; games; and traditional wisdom (eg, “water is life”) (13). Mihesuah and Hoover recently underscored the connection of food sovereignty to cultural knowledge, environments, and health (33).

The objective of this study was to describe our evaluation of a program designed to promote access to and integrate traditional foods, physical activities, and social support in semistructured ways into culturally and geographically diverse AI/AN communities. The Traditional Foods Project (TFP) provided modest funding and support to 17 AI/AN communities who designed their own interventions to meet the needs of their communities. Stories describing the innovative approaches based on traditional foods, culture, and history to prevent type 2 diabetes among TFP communities have been published elsewhere (13,14,26–30).

## Purpose and Objectives

The purpose of the TFP was to promote access to traditional foods, physical activity, and social support to address community health in AI/AN communities, particularly type 2 diabetes prevention. We sought to answer 2 questions. First, how do communities increase and sustain access to traditional healthy foods, physical activity, and social support to promote health and help prevent type 2 diabetes? Second, how do culturally and geographically diverse AI/AN communities, locally and in partnership with one another and a federal program, successfully evaluate interventions?

TFP objectives were to 1) support sustainable, evaluable ecological approaches to reclaim traditional foods, 2) encourage local practices to increase access to healthy traditional foods and physical activity, 3) revive and create stories of healthy traditional ways, and 4) integrate culture and history to promote community health and help prevent type 2 diabetes.

## Intervention Approach

The TFP evolved from the findings of earlier projects where traditional foods emerged as a way to promote health and help prevent type 2 diabetes. The Indian Health Service Tribal Leaders Diabetes Committee had suggested looking to tribal cultures to promote health and prevent type 2 diabetes among AI/ANs (13,14). These projects demonstrated that public health interventions are most effective when communities integrate their own cultures and history into local programs (13,14,38,39).

Community-based participatory research is the foundation of the TFP. In community-based participatory research, culture and context are legitimate foci for interventions (40), and partnering with communities in program design, evaluation, and reporting criteria is fundamental (40,41). Community-based participatory research methods were shaped by tribally driven participatory research (41) and framed by food sovereignty — the right of people to define their own policies and strategies for sustainable production, distribution, and consumption of food (34,42).

## Evaluation Methods

The TFP used both quantitative and qualitative evaluation methods. Mixed methods were critical to demonstrate which elements of each intervention worked (quantitative measures) and why and how communities became engaged across programs (qualitative measures). Honoring local knowledge and traditions, TFP partners catalyzed their communities such that farmers, health care providers, tribal leaders, subsistence gatherers, administrators, evaluators, and community members came together for the shared purpose of improving community health. Each TFP partner had a local coordinator and evaluator who developed community-supported programs and collected data in 3 domains: traditional foods, physical activity, and social support. All domain interventions were designed to improve health with the long-term goal of helping prevent type 2 diabetes.

### Setting and participants

The project began in 2008 with 11 tribes and tribal organizations and was expanded to 17 in 2009. The 17 TFP partners were culturally and geographically diverse. Each partner received $100,000 per year. In 2012, the Centers for Disease Control and Prevention’s Tribal Advisory Committee recommended a sixth year to increase capacity and sustain local efforts. Sixteen of 17 partners applied for and participated in the sixth year at the same level of funding (13).

The initial 11 TFP partners participated in all 5 years of data collection. Five of the 6 partners added later participated in 4 years of data collection. The remaining partner collected data for 3 years but did not participate in the final year.

### Procedures

The first year of the TFP focused on program and evaluation planning. Partners who launched the TFP in 2008 began gathering and reporting data in 2009, and partners who joined in 2009 began gathering and reporting data in 2010. Two 6-month data collection periods took place each year, resulting in 10 data collection periods and a sample size of 156 data points for each variable. Odd-numbered periods (T1, T3, T5, T7, T9) corresponded to data collected from October to March, including winter, when gardening was not possible in some communities. Even-numbered periods (T2, T4, T6, T8, T10) corresponded to data from April through September, including summer.

TFP partners reported on local activities and evaluation outcomes in 2 ways. First, partner evaluators entered local data addressing the 3 domains every 6 months into a shared data elements (SDE) tool developed jointly by the Native Diabetes Wellness Program and TFP grantee partners. The SDE was approved by the Office of Management and Budget (OMB no. 0920 0889). Having 2 collection periods during each intervention year allowed for seasonal analyses of partner activities. SDE data included quantitative information about overall numbers of activities across domains as well as numbers of participants, numbers of persons affected (through social media, local radio, and television), and brief qualitative descriptions of grantee partner activities. We gathered no individual health data for aggregate analysis because of cooperative agreement restrictions and the TFP focus on community health. Second, partners presented information on local program interventions, evaluation methods, and findings at TFP meetings either once or twice a year. Quantitative measures included number and size of gardens, weight and types of produce yields, and number of participants in organized physical activities. Qualitative data included stories used for teaching, descriptions of community responses, and examples of how culture and history were integral to TFP activities.

The Native Diabetes Wellness Program evaluation team aggregated and analyzed quantitative SDE data, with TFP partner program and period as the unit of analysis. We returned grantee-specific data to each partner along with aggregated SDE results after each data collection period. We also presented SDE data updates at every meeting.

We collected qualitative data by using the SDE and in other ways. The SDE used open-ended text fields of 50 to 250 characters to describe activities under general categories such as “gardening,” “health education,” and “measures of participant change.” Additionally, we encouraged TFP partners to collect other local data, such as stories. We also gathered partners’ written and oral comments during semi-annual or annual meetings and monthly conference calls.

### Intervention framework

The Native Diabetes Wellness Program did not prescribe methods of community intervention for the partners. Each TFP partner used various strategies aimed at behavior changes related to TFP goals. Overall intervention components were unique to each group. Components also differed over time, so the interventions implemented by a partner during, for example, the fourth period were likely different than its activities during the seventh period. Most partners engaged in 1 or more activities in each domain during each period.

Some program components affected more than 1 domain. For example, activities included in gardening or subsistence categories often involved physical activity and/or social support as well as traditional foods. For each activity, partners recorded which domain(s) they considered relevant to each project component.

### Measures

Each partner had flexibility to create and implement interventions consistent with local ways, based on local definitions of traditional healthy foods, physical activity, and social support. Traditional foods activities could include gardening, subsistence gathering, hunting, and fishing. Physical activity interventions focused on organized physical activities and places to conduct physical activity programs. We defined social support as any time local participants gathered to support each other, regardless of focus. General categories such as health education, health practices and policies, and storytelling were interventions across all domains.

### Quantitative data analysis

We stratified descriptive analyses by period and tabulated frequencies for categorical variables. Denominators used in percentages varied according to period, because the number of partners varied from 11 to 17. We tabulated numeric variables as counts, sums, measures of central tendency, and maximum values. The denominator used to calculated mean and median values for all 10 periods was 156. For many activities, TFP partners recorded the number of participants, the number of new participants, and the number of people affected in each period. We calculated total number of participants for individual activities across partner groups by period. We did not calculate sums of participants across periods, because we had no way to determine the amount of participant overlap between activities or across time.

We examined changes in the prevalence of all activities over time and used simple regression or the χ^2^ test for trend. The 156 data points collected were not independent, because groups were represented up to 10 times. We considered a repeated measures analysis but did not use it because the assumptions were not satisfied. For this reason, our analytic statistics examined trends but not relationships between outcomes. We used Stata version 13 (StataCorp LLC) for all analyses.

### Qualitative data analysis

The Native Diabetes Wellness Program reviewed SDE qualitative data after each data collection period. We also reviewed local qualitative data such as digital stories and other cultural applications of the 3 domains. We analyzed TFP partner evaluation forms and meeting and conference call notes for major themes and illustrative quotes. TFP partners reviewed the themes and quotes to ensure accuracy and intended voice.

## Results

### Quantitative findings

From 81.8% (9 of 11) to 94.1% (16 of 17) of TFP partners reported gardening activities during summer periods, and 58.8% (10 of 17) to 82.4% (14 of 17) during winter periods (Table 1). Garden types included school, community, family, and program gardens. Community gardens were reported by 37.5% (6 of 16) to 64.7% (11 of 17) of TFP partners for all periods (except T1, when the question about community gardens was not asked). The total number of all gardens increased over time, and ranged from a low of 13 gardens in T1 to a maximum of 510 gardens in T6 (n = 10 periods; controlling for season, *r* ^2^ = 0.85; coefficient = 25.9; *P* = .001). In T10, TFP partners reported 415 gardens, which covered an area of 28.4 acres, up from 206 gardens and 11.1 acres in T2 (the question about garden acreage was not asked in T1). Numbers of participants were highest for school gardens. For example, in T10, the 6 communities that had school gardens involved 3,017 people.

**Table 1:** Percentage of Grantee Partner Programs Engaging in Traditional Foods Project Activities by Period, Traditional Foods Project, October 2009–September 2014^a^

Activity	T1 (W) (n = 11)	T2 (S) (n = 11)	T3 (W) (n = 17)	T4 (S) (n = 17)	T5 (W) (n = 17)	T6 (S) (n = 17)	T7 (W) (n = 17)	T8 (S) (n = 17)	T9 (W) (n = 16)	T10 (S) (n = 16)	Median (T1–T10)
Planting and gardening activities	72.7	81.8	82.4	88.2	58.8	94.1	70.6	88.2	75.0	87.5	82.1
Community gardens	Not asked	54.6	41.2	52.9	41.2	64.7	52.9	64.7	37.5	50.0	51.4
Use of heirloom seeds	Not asked	Not asked	29.4	47.1	47.1	64.7	52.9	58.8	31.3	62.5	50.0
Composting	0	0	23.5	23.5	35.3	29.4	29.4	35.3	37.5	31.3	29.4
Healthy foods available in ≥1 venue	45.4	36.4	64.7	64.7	64.7	64.7	41.2	52.9	62.5	62.5	62.5
Health education activities/materials	81.8	100.0	88.2	94.1	82.4	85.4	76.5	82.4	81.3	87.5	83.9
Media outreach activities	36.4	45.4	64.7	64.7	52.9	64.7	64.7	58.8	56.3	56.3	57.6
New health policies and practices	45.4	45.4	41.2	47.1	58.8	41.2	41.2	41.2	43.8	31.3	42.5
Collaborate with other agencies	100.0	90.9	94.1	94.1	100.0	94.1	94.1	100.0	100.0	87.5	94.1
Organized physical activities	72.7	72.7	64.7	82.4	52.9	64.7	35.3	70.6	50.0	50.0	64.7
Participant change measured	54.6	45.4	47.1	47.1	41.2	47.1	47.1	41.2	25.0	50.0	47.1

Abbreviations: S, summer; W, winter.

a The “n” in each column refers to the number of partners, which is the same as the number of programs.

**Figure 1:**
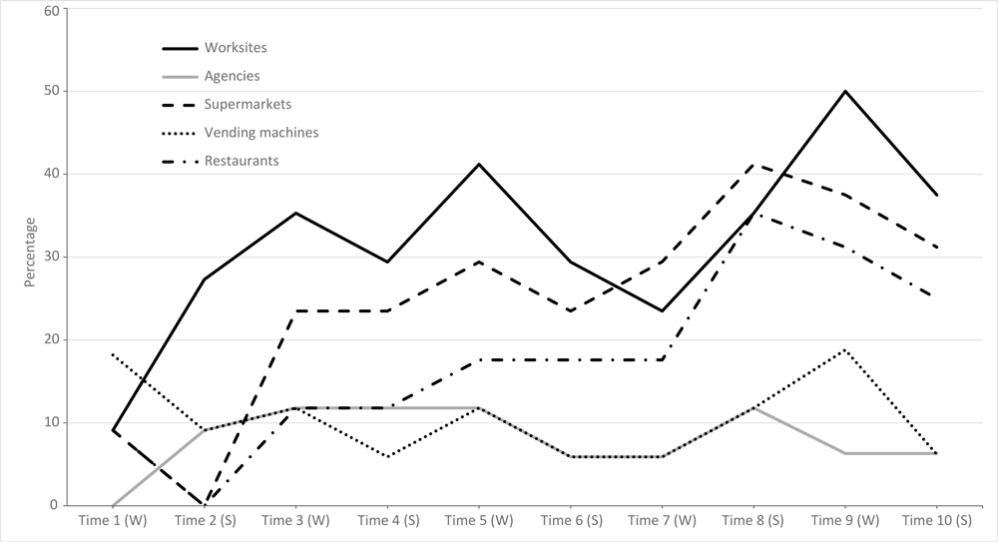
Percentage of partners reporting healthy food selections at worksites and other venues over time, Traditional Foods Project, October 2009–September 2014. Percentages are based on the following denominators: 11 partners participated during T1–T2; 17 partners during T3–T8; and 16 partners during T9–T10. Abbreviations: S, summer; W, winter.

Reporting on traditional healthy food outlets such as health fairs increased from 18.2% (2 of 11) in T1 to 68.8% (11 of 16) in T10. Access to healthy food at other venues also improved over time. By T10, nearly two-thirds of partners (62.5%; 10 of 16) reported that healthy food selections were available at 1 or more of the following venues: worksites, agencies, supermarkets, vending machines, and restaurants. More partners reported healthy food choices at worksites (37.5%; 6 of 16 in T10) and supermarkets (31.2%; 5 of 16) than at vending machines (6.3%; 1 of 16) and agencies (6.3%; 1 of 16) (Figure 1). Increases in the proportion of partners reporting access to healthy foods over time were significant for healthy foods available at restaurants (χ^2^ test for trend = 6.9; *P* = .008) and supermarkets (χ^2^ test for trend = 6.0; *P* = .01).

**Table:** 

Period	Worksites	Agencies	Supermarkets	Vending Machines	Restaurants
Time 1 (W)	9.1%	0%	9.1%	18.2%	9.1%
Time 2 (S)	27.3%	9.1%	0%	9.1%	0%
Time 3 (W)	35.3%	11.8%	23.5%	11.8%	11.8%
Time 4 (S)	29.4%	11.8%	23.5%	5.9%	11.8%
Time 5 (W)	41.2%	11.8%	29.4%	11.8%	17.6%
Time 6 (S)	29.4%	5.9%	23.5%	5.9%	17.6%
Time 7 (W)	23.5%	5.9%	29.4%	5.9%	17.6%
Time 8 (S)	35.3%	11.8%	41.2%	11.8%	35.3%
Time 9 (W)	50.0%	6.3%	37.5%	18.8%	31.2%
Time 10 (S)	37.5%	6.3%	31.2%	6.3%	25.0%

Storytelling was an important teaching activity for most TFP partners in every period; for example, 14 of 16 (87.5%) in T10 reported 1 or more storytelling activities. Most incorporated 1 or more types of storytelling (eg, narrative, digital, music) into program activities (Table 2). The highest proportions of storytelling activities were in the traditional foods domain, ranging from 52.9% (9 of 17) in T7 to 82.4% (14 of 17) in T3. Narrative storytelling activities were the most prevalent (mean, 58.3%, or 91 of 156 samples, over all periods), followed by digital (mean, 37.2%, or 58 of 156).

**Table 2:** Numbers of Stories and Participants, by Type of Media, Reported by All Traditional Foods Project Grantee Partners, Traditional Foods Project, October 2009–September 2014

Period	Narrative Stories		Digital Stories		Music, Plays, and Art Stories		Total	
	No. of Stories	No. of Participants	No. of Stories	No. of Participants	No. of Stories	No. of Participants	No. of Stories	No. of Participants
Time 1 (Winter)	73	787	14	47	19	740	106	1,574
Time 2 (Summer)	68	1,180	30	53	100	400	198	1,633
Time 3 (Winter)	155	1,088	132	68,416^a^	123	2,995	410	72,499
Time 4 (Summer)	193	1,495	34	109	136	1,668	363	3,272
Time 5 (Winter)	146	1,899	31	117	7	265	184	2,281
Time 6 (Summer)	82	1,319	24	141	37	498	143	1,958
Time 7 (Winter)	130	7,715	24	434	39	1,052	193	9,201
Time 8 (Summer)	101	1,717	18	94	113	771	232	2,582
Time 9 (Winter)	145	7,383	40	325	34	490	219	8,198
Time 10 (Summer)	112	10,975	55	142	108	899	275	12,016

a Includes the number of people reached through social media.

Most TFP partners reported health education activities for each period (range, 76.5% [13 of 17] in T7 to 100% [11 of 11] in T2). Individual TFP partners reported implementing up to 180 health education activities in a 6-month period (T10) and involving a maximum of 10,900 participants (T5).

TFP partners reported implementing new health practices (including behaviors, resolutions, policies, and other practices not done before) during each 6-month period at an overall rate of 43.6% (68 of 156 total data points), with a maximum of 58.8% (10 of 17 partners) in T5. The total number of new health practices over all groups for any 1 period ranged from 12 (T1) to 78 (T5). As an example, 1 partner reported that their after-school/summer camp implemented a policy that included not having sugar-sweetened beverages and candy available for purchase. In another, Head Start organizations added physical activities, gardening, and a health education curriculum to their programs.

Most partners reported including organized physical activities in their programs (overall average for all periods, 60.9%, or 95 of 156). As many as 7,500 participants were involved in organized physical activities for an individual TFP partner during 1 period (T3). Examples of organized activities included traditional games such as stickball, fun runs, restoration work, canoeing, and dancing.

Partners measured participant changes such as weight loss, improved levels of physical activity, and healthy food choices in 69 of 156 data points recorded during 10 periods. In most periods, almost half (median, 47.1; 8 of 17) of partners were measuring participant change in 1 or more domains (Table 1). Numbers of participants who made changes increased from T1 to T10 for each of the 3 domains, with a maximum at T6 in physical activity (n = 1,388 participants) and social support (n = 1,950 participants) and a maximum at T8 in traditional foods (n = 2,152 participants).

Almost all TFP partners reported collaboration with other agencies in all 10 periods. The proportion of partners reporting at least 1 type of collaboration ranged from 87.5% (14 of 16 in T10) to 100% (11 of 11 in T1; 17 of 17 in T5 and T8; and 16 of 16 in T9). Collaboration was reported most often in the traditional foods domain (89.7% of partners; 140 of 156 overall for the 10 periods), and by most grantees in the physical activity (59.6%; 93 of 156) and social support (56.4%; 88 of 156) domains. Resources shared included staff (71.8%; 112 of 156), space (60.3%; 94 of 156), educational materials (59.6%; 93 of 156), traditional foods (55.1%; 86 of 156), marketing materials (44.9%; 70 of 156), and financial support, such as vouchers (40.4%; 63 of 156).

Media outreach events and materials were described in 103 of 156 (66.0%) reports. In T10, 16 partners conducted 308 media outreach events, developed and distributed 9,264 materials, and affected 31,400 people with media materials. In T1, when programs were just getting started, media was even more commonly reported. The 11 partners reported 1,614 media outreach events at which 77,523 media materials were distributed and 278,235 people affected.

In T10, 56.2% (9 of 16) of TFP programs reported implementing environmental changes in 1 or more domain areas that were designed to be sustainable. Sustainability was also evidenced in activities reported in every period, eg, planting and gardening, particularly community gardening (ranging from 37.5% [6 of 16] to 64.7% [11 of 17] partners). Other examples of sustainable environmental changes included using heirloom seeds, composting, developing health education activities and materials, implementing media outreach activities, implementing health policies and health practices, and collaboration with other agencies (Table 1) .

### Qualitative findings

Qualitative data portrayed the role of traditional foods in ways quantitative data could not. The data describe partner perspectives about traditional foods, how well the TFP worked, and why. Results indicate that grantee partners embraced the TFP’s community-based, tribally driven approach. Themes and quotes underscore quantitative findings, such as participation, collaboration, and number of gardens (Table 3).

**Table 3:** Themes and Theme-Specific Quotes From Traditional Foods Project Grantee Partners, Traditional Foods Project, October 2009–September 2014

Themes	Theme-Specific Quotes
Traditional knowledge and grassroots	• Focusing on traditions is where connections are made. • The focus on tradition and culture is the basis of this project. This is why it’s so important at the grassroots. Otherwise it is “just funding with a Native design.” • Traditional ecological knowledge guides the way in balance with western science. • Top-down models don’t work well. What works is “on the ground.” • Our elders get this. • What we’ve done here is show that tribes can do what works for them. • It is brave to define truth; what tribes know is important. • Traditional foods are probably one of the most important elements in any Native American/Alaskan Native’s culture. • This project is not a temporary spark for this community, but a lifestyle deeply rooted in our culture. We must continue this effort to eat healthily and keep moving. We must all lend a hand and be part of a voice in keeping our people healthy. • Being on the land allows for learning more deeply through host grantee’s history, culture, and traditions that shape their food system. • No matter how we design our data collection instruments, only members of our communities can make true assessments of the impacts and outcomes of an intervention. • Participation in the sacred may not be a requirement of a federal job, but is almost always a requirement of working effectively with tribes.
Connections to health	• We are reconnecting land and water with health. • Traditional foods have become the way to talk about health. • Traditional foods provide an alternative to high-cost, low-quality foods offered in many tribal communities by convenience and grocery stores. • Type 2 diabetes is part of intergenerational trauma. • Diabetes and related diseases have been with our communities for 1 or 2 generations, and in many cases, traditional foodways that can prevent, treat, and cure these diseases have been gone for that amount of time or longer. It’s going to take at least that long to meaningfully address these diseases.
The power of stories and storytelling	• Such great stories are told through presentations about changing a person’s life or what an elder said. Can we capture these compelling moments? The reporting we do is so rich with stories. • At the time we incorporated the Eagle Books to our program, what helped was how relatable the books were to our kids. When the lessons were in story form, the students stayed interested in the health messages we were trying to get across. • Each traditional food procurement involves not only the return to a healthier subsistence diet but the physical activity associated with growing or obtaining the food. It also requires a sharing of stories and knowledge about how it is prepared or how that food shaped the lives of the ancestors. Such stories span generations.
Community engagement	• Youth are being engaged in learning traditional knowledge and helping their people. • This project has community members working together who wouldn’t ordinarily have the opportunity. • It took 3 years for our tribe to gain full community buy in. Volunteers are abundant resources now. • Tribal members are participating in Traditional Foods advisory boards and food policy councils. • Traditional foods projects are supporting traditional knowledge. • We envision these garden spaces to be more than just a place to grow food, but also a place where community members can gather. • Our community sees the change this program has made — this is extraordinary for our people who are so often hopeless that they can change their situation. Not only that, they stood as witnesses to the successes felt in the larger community of TF partner sites. • It is exciting that traditional foods are resonating with youth. These spaces for intergenerational community are ever-valuable in a modern world where young people communicate mostly through digital mediums.
Knowledge sharing and gratitude	• Food is good medicine. Traditional foodways include responsibility, giving thanks, and sharing. • Sharing traditional foods and cultural practices is the foundation of our program. • We got this idea from [name of TFP partner]. Sharing across the country and across the room is meaningful and helpful. • The emergence of the theme of how spiritual our traditional foods are — the big picture. I think we all have known this, but it was amazing to me that this emerged at a meeting and flowed freely. This was a refreshing time for me. • My great aunt used to tell me that our foods taste better when we share them. In this case [TFP meeting] we shared them with our own local Native community and with all of the other communities represented. The good feelings were palpable and dinner was delicious. • It is great to expand to Indian Country and share what we have learned. • Thank you for giving me one of my life’s biggest blessings — the chance to be part of this group. I ask the Creator to bring us all together again. • I feel like part of a large family of inspiration and affirmation.
Flexibility to do what works	• The flexibility to do what works is invaluable. In our experience with federal programs, we are not doing what you want us to do most of the time. With this program, we are doing exactly what you want us to do! • Policy seems most effective if it comes from the grassroots. Policy, or practice, that allows people to have choice is bridged with traditional practice. • Practice-based evidence is what we are doing. • There are impacts the TFP is not measuring like substance abuse and environmental health. • We are recognizing tribal sovereignty. • Do I really get to do this?
Program sustainability	• It took a long time for our people to get sick. Sustainability will not happen in 4 to 5 years. • It would be a shame not to continue these programs without giving them a chance to show the impact of this work. • Just imagine what we could do with another cycle! We have shared and borrowed so much from each other already. We could deepen this effort. • The Traditional Foods Project has made an impact on this reservation. Maybe a decade or two ago there were only a handful of gardens here. Now we till over 50. That is equivalent to almost one-fourth of the households raising their own garden.

The following examples of tribal partner experiences further illustrate the 7 main themes. The examples usually include more than 1 theme, demonstrating not only their interconnectedness but also how difficult it was for us to separate them.

**Figure 2:**
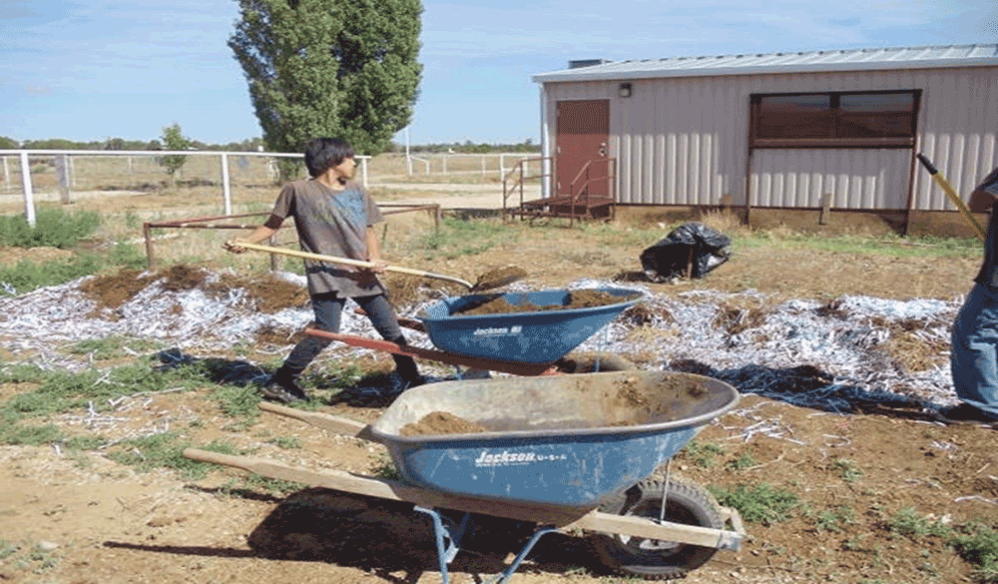
The compost pile was created to increase produce yield in the community garden, Traditional Foods Project, October 2009–September 2014. Compost materials included paper and coffee grounds provided by tribal leaders, Ramah Navajo, 2011. Photo courtesy of Randy Chatto.

**Traditional knowledge and grassroots.** Local elders remarked that corn did not grow very high in their community’s desert soil. The TFP coordinator took a course to become a Master Composter, balancing traditional ecological knowledge and western science. He created a compost pile to be used in the community garden to increase produce production. In addition to other compost materials, tribal leaders provided an endless supply of discarded paper and coffee grounds (Figure 2). Community members, particularly the elders, were impressed with how tall the corn grew and marveled at the large yields of harvested produce from the garden.

**Connections to health.** TFP partners inspired the title of this article. “Traditional foods have become a way to talk about health” was a thread in every discussion. Partners could not underscore enough that chronic disease is deeply connected to social determinants of health, such as historical trauma, adverse childhood experiences, and loss of traditional foodways. The way to reclaim health, they said, is to reconnect with the land, water, traditional foodways, and all that they mean.

**The power of stories and storytelling.** Narrative stories — oral tradition — were most prevalent compared with other types of stories reported (Table 2). However, TFP partners enthusiastically produced digital stories after learning from another partner how to create them. In turn, they shared the skill with their own people. One story was by a young rapper who had struggled with identity and substance abuse. He “found himself through connection with the earth” in the community garden. He created his digital story to welcome all partners, skillfully rapping their names, at a TFP meeting.

**Community engagement. **Meetings hosted by TFP partners provided settings for sharing traditional foods, cultural ways, and physical activity. One of the most anticipated activities was the traditional game of stickball. The community was invited to participate or observe (and cheer). Stickball literally created a level playing field, where TFP partners, Native Diabetes Wellness Program team members, and community members, women against men, enjoyed a physically strenuous, humor-filled game.

**Knowledge sharing and gratitude**. Dynamic exchange of knowledge demonstrated partners’ engagement with each other. They shared skills (how to create digital stories), traditional foods (meeting hosts always prepared a feast), and gifts (heirloom seeds, wild rice). Partners were grateful for being able to openly express the meaning of traditional foods and spend time together.

**Flexibility to do what works.** At the request of grantee partners, we held a discussion on health policy in the second year of the TFP. Partners stated that measuring health policy only was unacceptable: “written policies tell people what to do.” Health practices, however, “are chosen by the people because they are good ideas and reflect traditional knowledge.” Subsequently, we measured both health policies and health practices.

**Program sustainability.** TFP partners regularly addressed sustainability, particularly toward the end of the TFP. Most partners (11 of 17) sustained some or all activities after the TFP ended in September 2014. Partners secured funding from tribal councils, university partnerships, state and county health departments, federal agencies, or nonprofit organizations (13). 

## Implications for Public Health

The TFP’s challenge was to answer 2 questions: How do communities increase and sustain access to traditional healthy foods, physical activity, and social support to promote health and help prevent type 2 diabetes? And, how do we, in partnership with one another, successfully evaluate community-based interventions?

Increasing and sustaining access to traditional foods depends on strong local support, collaboration, and traditional knowledge (25–30,33). Grantee partners believed that traditional foods programs can be sustained if the following conditions are met:

• First, human and financial resources are necessary. A local natural leader, knowledgeable about traditional foods and supported financially, is vital.

• Second, tribal leadership support is needed. Where tribal leadership was not supportive, TFP programs were less productive. In contrast, strong backing by tribal leadership contributed to project endurance.

• Third, sustainability is likely when programs are relevant and meaningful. Local decisions about program content, including what constitutes traditional foods, are critical.

• Fourth, collaborating with programs that have related goals strengthens community infrastructure. Partners noted that, over time, other programs sustained activities originated by the TFP.

• Fifth, communities with few resources need time to grow infrastructure. Among TFP partners, small communities demonstrated change quickly but, without strengthened infrastructure, changes were temporary.

Tribally based health promotion efforts to address access to traditional foods in Indian Country are described in the 2015 report, *Feeding Ourselves* (34). Our conclusions are consistent with those described in the report in the section “Case studies: lessons learned and challenges faced by grassroots, nonprofit and tribal food access and health innovators.” As an example, the Communities Creating Healthy Environments program addressed childhood obesity by changing communities rather than focusing on individual behaviors, incorporating aspects such as food inequity, safe places for play, and the social environment. The program noted not only the need for local partners but also the need for ongoing support to “implement victories, consolidate gains, and plan next steps” (34).

For our project to be successful, forging trust among TFP partners and the Native Diabetes Wellness Program was paramount (40). Further, equal funding, regardless of community size, gave every program equal voice. In the end, relationships were everything (13,14,26–30,33,34,40).

TFP data did not include aggregated health measures for individual participants (eg, weight change over time) because of funding restrictions and the focus on environment and community. Future TFPs would benefit from tracking changes in individual health outcomes across communities. Collecting local health data may be challenging, however, because of the sensitivity of personal health information. Tribal nations are particularly cautious about sharing personal health data because of their experiences with data misuse (40,43). This history underscores the critical importance of a tribally driven participatory approach (41), where tribes steer the agenda in partnership with the funding entity to develop the program, choose local and aggregate evaluation measures, and select outcomes.

Population sizes and geography varied widely among participating communities. TFP partners used intervention combinations designed for local conditions that could not be directly compared across sites. Environmental factors also made it difficult to compare certain interventions, such as gardening, because some communities had longer growing seasons than others.

We did not conduct bivariate analyses of the relationships between interventions and outcomes (eg, gardening activities and health policy changes). The project was not designed to imply such causal relationships.

It is methodologically challenging to distinguish effects of a particular program when multiple agencies work together. However, working collaboratively makes any single program, and subsequent community infrastructure, stronger.

The TFP addressed physical activity, social support, and healthy diet, factors associated with individual and community health. Partners developed local programs, framed in local cultural, historical, and environmental contexts, which included social determinants of health. Activities incorporated traditional ecological knowledge and western science, illustrating the integral relationship of traditional foods with community history, culture, and health. The TFP demonstrated that tribally driven programs, guided by traditional knowledge, can facilitate access to traditional foods as part of community health interventions to address chronic disease.

“Traditional ways of knowing” have, for generations, linked physical and spiritual health to traditional foods (9,25–30,34,44). The concept is far from new. What is new is the burgeoning food sovereignty movement that reclaims traditional foods in relation to tribal sovereignty, food security and, in this instance, public health. Traditional foods have become, once again, a way to talk about health.
